# Supplementary figures and images for: Automated ELISA On-Chip for the Detection of Anti-SARS-CoV-2 Antibodies
Source: Sensors (Basel). 2021 Oct 13;21(20):6785. doi: 10.3390/s21206785 (PMC8539637; doi:10.3390/s21206785)

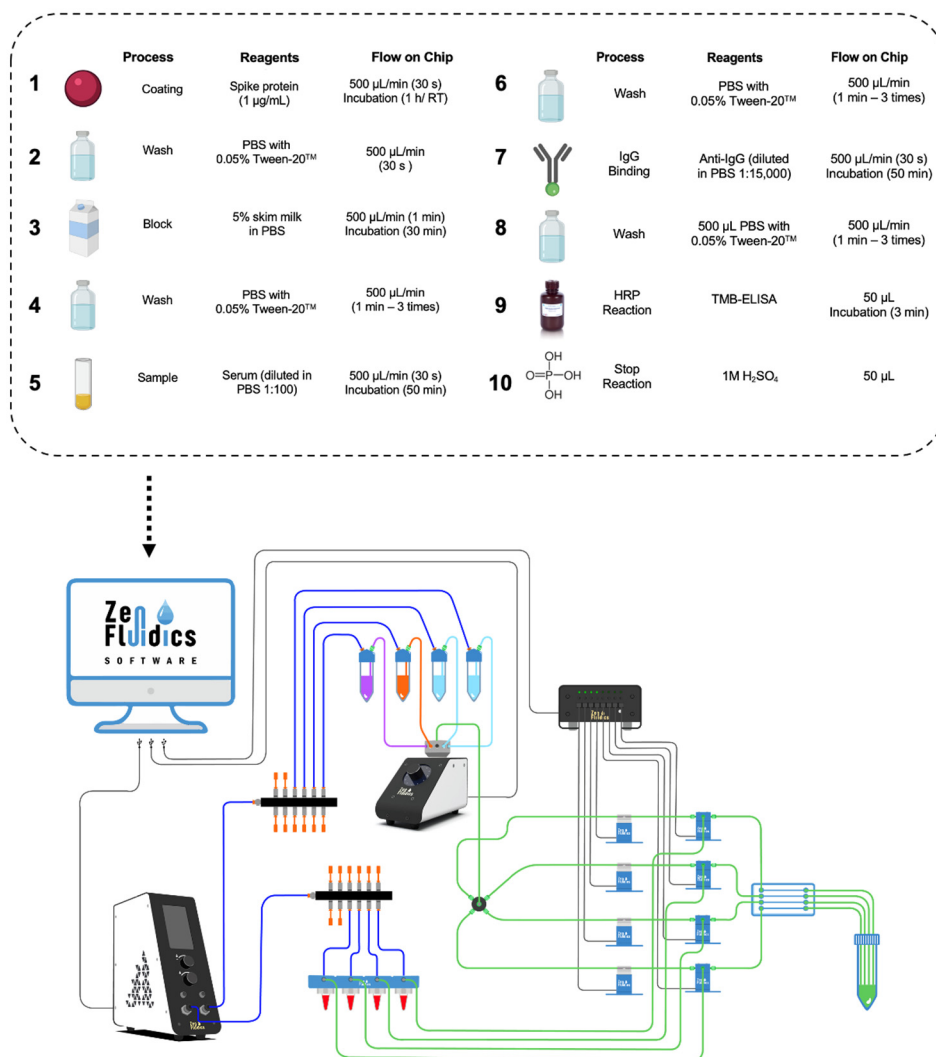

**Figure S1.** Established protocol and experimental setup of our automated ELISA on-chip.

Supplement: Supplementary file 1 [file sensors-21-06785-s001.zip › sensors-1346583-supplementary.pdf]
